# Tachycardia in Post-Infarction Hearts: Insights from 3D Image-Based Ventricular Models

**DOI:** 10.1371/journal.pone.0068872

**Published:** 2013-07-02

**Authors:** Hermenegild Arevalo, Gernot Plank, Patrick Helm, Henry Halperin, Natalia Trayanova

**Affiliations:** 1 Department of Biomedical Engineering, Johns Hopkins University, Baltimore, Maryland, United States of America; 2 Institute of Biophysics, Medical University of Graz, Graz, Austria; 3 Medtronic Inc., Minneapolis, Minnesota, United States of America; 4 Department of Medicine, Johns Hopkins University, Baltimore, Maryland, United States of America; University of Otago, New Zealand

## Abstract

Ventricular tachycardia, a life-threatening regular and repetitive fast heart rhythm, frequently occurs in the setting of myocardial infarction. Recently, the peri-infarct zones surrounding the necrotic scar (termed gray zones) have been shown to correlate with ventricular tachycardia inducibility. However, it remains unknown how the latter is determined by gray zone distribution and size. The goal of this study is to examine how tachycardia circuits are maintained in the infarcted heart and to explore the relationship between the tachycardia organizing centers and the infarct gray zone size and degree of heterogeneity. To achieve the goals of the study, we employ a sophisticated high-resolution electrophysiological model of the infarcted canine ventricles reconstructed from imaging data, representing both scar and gray zone. The baseline canine ventricular model was also used to generate additional ventricular models with different gray zone sizes, as well as models in which the gray zone was represented as different heterogeneous combinations of viable tissue and necrotic scar. The results of the tachycardia induction simulations with a number of high-resolution canine ventricular models (22 altogether) demonstrated that the gray zone was the critical factor resulting in arrhythmia induction and maintenance. In all models with inducible arrhythmia, the scroll-wave filaments were contained entirely within the gray zone, regardless of its size or the level of heterogeneity of its composition. The gray zone was thus found to be the arrhythmogenic substrate that promoted wavebreak and reentry formation. We found that the scroll-wave filament locations were insensitive to the structural composition of the gray zone and were determined predominantly by the gray zone morphology and size. The findings of this study have important implications for the advancement of improved criteria for stratifying arrhythmia risk in post-infarction patients and for the development of new approaches for determining the ablation targets of infarct-related tachycardia.

## Introduction

Ventricular arrhythmia, the pathogenesis of which results from abnormal impulse propagation in the heart, is a leading cause of death in the industrialized world [1]. Ventricular tachycardia (VT), a life-threatening regular and repetitive fast heart rhythm, frequently occurs in the setting of myocardial infarction (MI). Implantation of a cardioverter-defibrillator (ICD) is the most effective measure for preventing lethal arrhythmias in post-MI patients. Despite this critical survival benefit, ICD therapy is costly and can be associated with procedural complications, infections, device malfunctions and diminished quality of life [2]–[5]. Current clinical criteria for identifying ICD candidates for the primary prevention of sudden cardiac death rely almost exclusively on a nonspecific reduction in global left ventricular function (ejection fraction≤35%). Only 5% of patients who meet this criterion and thus undergo device implantation receive life-saving appropriate defibrillation shocks. Development of patient selection criteria of higher specificity for arrhythmia risk is currently hindered by the lack of understanding of the relationship between the complex structural remodeling in MI and infarct-related VTs.

It has been shown that magnetic resonance imaging (MRI) with late gadolinium enhancement (LGE) can be used to detect infarct location and distribution in animal models [6], [7] and humans [8], [9]. Recently, the peri-infarct (border) zones surrounding the necrotic scar, also known as gray zones (GZ) based on their appearance as regions of intermediate intensity in the LGE-MRI scans, have been shown to correlate with post-MI mortality [10], clinical VT [11], and VT inducibility [12]. Histological studies have shown these GZ regions to be a heterogeneous mix of viable myocardium and necrotic scar [13]. Animal experimental evidence has implicated the GZ as the arrhythmogenic substrate in MI [14]; Ashikaga et al demonstrated that in infarcted swine hearts, reentrant circuits were anchored to strands of viable myocardium positioned over intramural scars [15]. However, it remains unclear how infarct-related VTs relate to the specific GZ distribution and size. Addressing this question would provide an impetus to the development of improved criteria for stratifying arrhythmia risk in post-MI patients.

The present study aims to address this question. We employ a sophisticated high-resolution MRI-based model of the infarcted canine ventricles to explore the infarct-related VT circuits as well as the relationship between the locations of the VT organizing centers and GZ distribution. The approach allows us to determine how the specific region of infarct (GZ and/or scar) maintains the VT circuits, and whether VT inducibility depends on GZ size. We also address the issue of the impact of the level of structural heterogeneity within the GZ on infarct-related VT inducibility and reentrant circuit morphology.

## Methods

### Baseline Post-MI Canine Ventricles Model: Representation of Infarct Morphology

**Figure 1:**
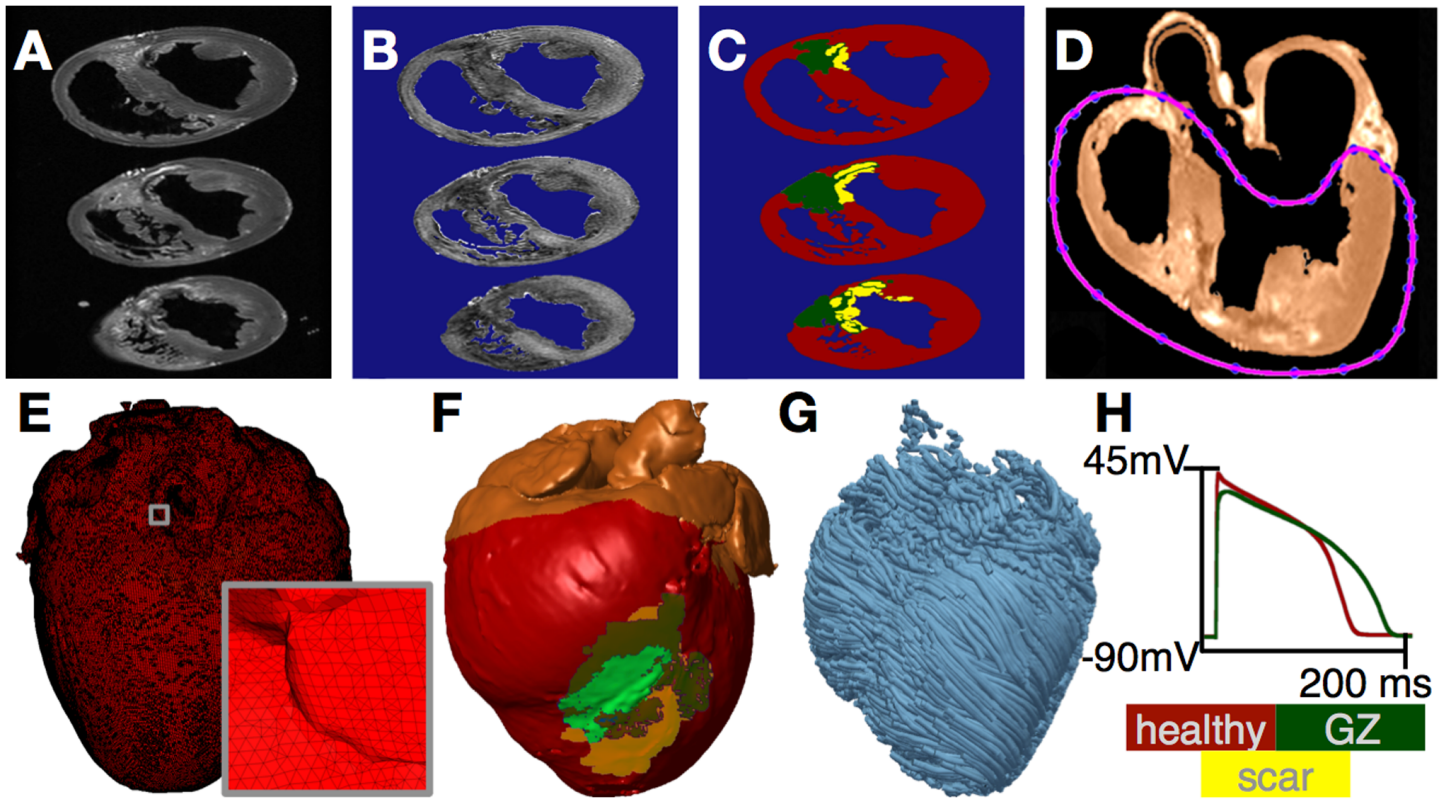
Model generation. (**A**) Ex-vivo MRI scan of an infarcted canine heart. (**B** FA maps as calculated from the DT-MRI, brighter color corresponds with higher FA value. (**C**) Segmentation of the images into healthy myocardium, GZ, and scar. (**D**) Separation of atria from ventricles. (**E**) Finite element mesh. (**F**) 3D model of the canine heart with the epicardium rendered semi-transparent. (**G**) Streamline representation of fibers obtained from DTMRI. (**H**) Action potentials of healthy myocytes and GZ cells.

To understand comprehensively how VT is sustained in the post-MI heart, we used a biophysically-detailed model of an individual canine heart post-MI, reconstructed from high-resolution ex-vivo MRI and diffusion tensor (DT)-MRI scans (Fig. 1A–B). Figure 1C–G presents the generation of the geometry/structure of the canine ventricular model. Detail regarding the image acquisition and partial description of the model reconstruction has been published previously [16]. Briefly, a canine heart ∼4 weeks post-infarction was scanned (ex-vivo MRI and DTMRI) at a resolution of 350×350×800 µm^3^ and interpolated, using cubic splines, to a resolution of 200×200×200 µm^3^, to ensure accurate segmentation (Fig. 1A and B). A level set method was applied to the MR image stack to separate the myocardium from the surrounding suspension media (Fig. 1C). Once an accurate reconstruction of the myocardium was obtained, the ventricles were delineated from the atria. To perform this step, a closed spline curve was fitted through landmark points placed around the ventricles and along the atrio-ventricular border; all voxels that belonged to the tissue inside the closed curve were marked as ventricular (Fig. 1D). The identification of landmark points was performed manually for a number of slices that were evenly distributed in the image stack. The landmarks for the remaining slices were obtained by linearly interpolating the manually identified points.

After the separation of the ventricles from the atria, infarct tissue was labeled. Since the high-resolution ex-vivo MRI acquisition protocol did not involve the use of contrast agents such as gadolinium, we developed a two-step methodology that combined information from both the DTMRI and the structural MRI scans to segment out the two infarct zones, the scar and the electrically-remodeled gray zone (GZ). First, the effective diffusion tensor (D_eff_) at each pixel was calculated from the DTMRI dataset as done by Basser et al. [17]. Next, fractional anisotropy (FA) values were calculated from D_eff_ to quantify water diffusivity at each pixel in the ventricles as described in [18] (Fig. 1B). FA values range from 0 to 1, with 0 indicating isotropic diffusion and 1 indicating diffusion restricted along one axis only. It has been shown that remodeling within the infarct (both in scar and in GZ), characterized with increased presence of cells such as necrotic tissue, collagen, macrophages, and fibroblasts, results in a lower FA value compared to the normal myocardium [18]. Thus, level-set thresholding of the FA images was used to discriminate infarcted tissue from the normal myocardium. In the second step, the infarct region was further subdivided into inactive scar and GZ by thresholding the structural MR image based on the intensity values. Scar tissue appears in the MRI scans as central dark regions surrounded by hyperenhanced regions [19]. Thus, pixels with high (>75%) and low (<25%) gray-level intensities within the low-FA region were designated as electrically-inert scar, while pixels with intermediate intensities (≥25% and ≤75%) were designated as GZ. The resulting infarct segmentation revealed strands of GZ tissue penetrating the electrically inert scar tissue, forming channels (Fig. 1C and 1F). The volumes of the segmented ventricular regions were 123.7 cm^3^, 5.3 cm^3^, and 5.0 cm^3^ for the entire ventricular myocardium, the scar, and the GZ, respectively.

A finite element computational mesh was constructed directly from the segmented images using a published approach [20]; it preserved the fine geometric detail of the ventricles and of the different zones within the infarct (Fig, 1E–F). The mesh of the infarcted canine ventricles consisted of 3,177,732 nodes (2,185,112 nodes in the ventricles) and 3,981,196 (2,603,624 in the ventricles) mixed-type elements [21] with an average element edge length of 396 µm. Finally, fiber orientations were assigned to each element by calculating the primary eigenvector from the corresponding DT-MRI (Fig. 1G). A similar approach for ex-vivo MRI-based heart reconstruction has been used in our recent studies [22]–[24].

### Baseline Post-MI Canine Ventricles Model: Representation of Electrophysiological Properties

Mathematical description of cardiac tissue was based on the monodomain representation [25]. The scar was modeled as an insulator (collagen). Anisotropic conductivities in the normal myocardium were assigned values as in [26], matching canine conduction velocities reported by Roberts et al [27]. Within the GZ, the transverse conductivity was decreased by 90% to match reported conduction velocities [15], thus reflecting connexin 43 (Cx43) downregulation and lateralization [28].

Membrane behavior in the canine ventricular model was represented by the Luo-Rudy dynamic model (LRd) [29], typically used as a generic mammalian membrane model in numerous studies of arrhythmias and spiral wave behavior [30]–[33]; the LRd is considered of medium-to-low complexity and is a reasonable trade-off in large scale models such as our whole canine heart. The same membrane model was used, with modifications based on experimental data, to represent the electrophysiology of GZ cells. Specifically, patch clamp studies using cells harvested from the epicardial border zone of infarcted canine hearts have reported a reduction in peak sodium current to 38% of the normal value [34]; in peak L-type calcium current to 31% of normal [35]; and in peak potassium currents IKr and IKs to 30% and 20% of the normal [36], respectively. These modifications were implemented in the LRd model to obtain a GZ action potential; the latter was characterized, consistent with experimental recordings [37], with 25% longer action potential duration, 31% smaller upstroke velocity, and 32% lower peak action potential amplitude compared to that of the normal myocardium (Fig. 1H).

### Additional Post-MI Ventricular Models to Explore the Role of GZ Size and GZ Level of Heterogeneity in VT Morphology

To explore the role of GZ size in VT dynamics, the baseline infarcted canine model described above was modified to generate infarcted canine ventricular models with decreased GZ volumes. The rationale for this step was that canine hearts have an extensive epicardial GZ [38], while human hearts have typically intramural infarcts with smaller size GZs. The decrease in GZ volume thus allowed us to obtain insight into human infarct-related VT.

We used the software ImageJ (http://imagej.nih.gov/ij/) to perform the operation “3D morphological erosion”, which decreases the object volume without changing the 3D structure [39]. The operation was performed such that only the position of the boundary between GZ and normal tissue was relocated, while the boundary between scar and GZ remained unchanged. We decreased GZ volume to reach values found in arrhythmogenic human hearts [12]. The operation was performed 3 times to obtain 3 models with GZ volumes that were 64%, 37%, and 15% of the baseline model.

In the baseline model and the models above, the GZ was represented as a homogeneous region of averaged remodeled electrophysiological properties. Presence of patches of scar in the GZ was not explicitly represented, but rather via the changes in conduction velocity. Histological examinations of infarcted tissue have shown that voxels identified as GZ from MR scans correspond to microscopically heterogeneous mixtures of viable myocardium and infarct scar [13]. We investigated whether explicit representation of scar patches in the GZ would affect the calculated VT morphology. To do so, we generated several models with heterogeneous GZs, where micro-regions of scar tissue and normal myocardium were randomly distributed throughout the GZ at varying degrees (from 10% to 90% of GZ volume in 10% steps, 18 ventricular models altogether).

### Simulation Protocol and Analysis

All simulations were performed using the software package CARP (CardioSolv, LLC) on a parallel computing platform; the numerical methodology has been described in previous publications [25], [40], [41]. We use sophisticated solver techniques, which ensure high efficiency, accuracy and stability of our numerical solutions for computational grids of the size of the canine heart. Our spatial and temporal discretization steps were chosen specifically, after extensive testing, to achieve maximum performance while ensuring accuracy and convergence.

To examine the arrhythmogenic propensity of the infarcted ventricular models, programmed electrical stimulation (PES), similar to protocols used for clinical evaluation of post-MI patients, was simulated [42]. The models were paced from an endocardial location for 6 beats (S1) at a cycle length of 300 ms followed by a premature stimulus (S2) initially given at 90% of S1 cycle length. The timing between S1 and S2 was progressively shortened until VT was induced. If VT was not induced, a second premature stimulus (S3) was delivered after S2. PES was performed from 27 different endocardial sites to ensure that all possible VT morphologies arising from the infarct geometry will be evaluated.

Pseudo-ECGs were generated by taking the difference between the calculated extracellular potentials at two points in an isotropic conductive medium surrounding the heart. The two points were near the base of the heart and separated by 18 cm, and the line connecting them was perpendicular to the apex-base axis running through the LV mid-wall, septum, and RV mid-wall. The extracellular potentials were calculated using the integral equation by Gima et al [43].

Infarct-related VT dynamics was analyzed by determining the scroll-wave filaments, which are the organizing centers of reentrant activity [44]. The filaments were calculated by first converting transmembrane potential data into spatial phase angle maps which were then processed to identify regions where the phase was undefined [45]. Each filament was classified into I or U type based on where the filament endpoints were located. Filament endpoints could be located at any surface that delineated inexcitable and excitable tissue; in the infarcted ventricular models these would be the epicardium, endocardium, and the scar. I-type denoted filaments with endpoints located at two different surfaces and U-type filaments had endpoints on the same surface.

## Results

### Location of the 3D Organizing Centers of Infarct-Related VT

**Figure 2:**
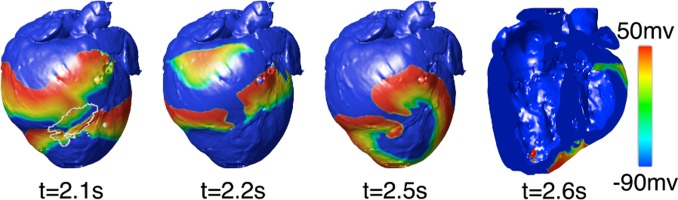
VT induction in the canine heart. Typical transmembrane potential maps during PES-induced VT (GZ on epicardium outlined in white).

VTs were induced in the baseline canine post-MI ventricular model following PES from 8 out of the 27 pacing sites. All VTs persisted for the entire 2 s of simulated time interval. For all VTs induced, reentry initiation took place within the GZ. Figure 2 presents epicardial transmembrane potential maps depicting the events leading to reentry initiation for PES from an endocardial site near the LV apex. The GZ (outlined in white on the epicardium) exhibited slowed conduction and longer recovery time compared to the surrounding healthy tissue (Fig. 2, 2.1 s). This resulted in conduction block (Fig. 2, 2.2 s), wavebreak, and the formation of reentry (Fig. 2, 2.5 s). For all PES sites resulting in VT induction, the reentrant circuit manifested itself as a figure-of-eight pattern on the epicardium and breakthrough(s) on the endocardium (Fig. 2, 2.6 s).

**Figure 3:**
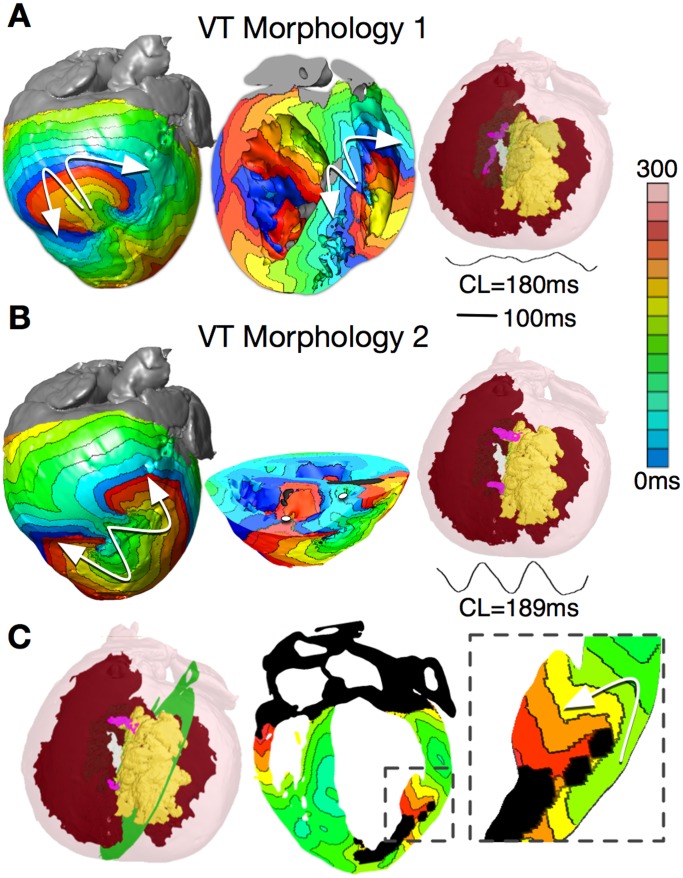
Reentry morphologies during post-MI VT. (**A**) VT morphology 1: Activation maps on the epicardium and in a long-axis cross-section of the ventricles, revealing figure-of-eight reentries on the epicardium and on the RV endocardium. VT is sustained by two I-type filaments (pink lines) located within the GZ with endpoints on the epicardium and RV endocardium. (**B**) VT morphology 2: Activation maps on the epicardium and in a short-axis cross-section of the ventricles, revealing figure-of-eight reentry on the epicardium and two breakthroughs on endocardium (white dots). Reentry was organized around two I-type filaments with endpoints on the epicardium and scar (pink lines). The pseudo-ECGs associated with both VT morphologies are also shown. (**C**) Activation map showing apparent reentry around a scar distal from filaments.

The VT morphologies induced from the 8 pacing sites were not all unique. Comparison of pseudo-ECGs demonstrated two distinct VT morphologies. The first VT morphology resulted from PES at two sites, both on RV, and had an average cycle length of 190±14 ms. The reentrant circuit was a figure-of-eight pattern on the epicardium and RV endocardium (Fig. 3A). For this VT morphology, the reentry revolved around two I-type filaments with endpoints at the epicardium and RV endocardium (Fig. 3A, pink lines). The filaments were fully contained within the GZ and the endpoints remained in the same locations for the duration of the VT.

The second VT morphology resulted from PES at six LV endocardial sites. The average cycle length, 222±17 ms, was longer than that of the first VT morphology. The figure-of-eight reentry on the epicardium had a direction of rotation (chirality) opposite to that of the first VT morphology, and was manifested as breakthroughs on the LV and RV endocardial surfaces (Fig. 3B). This was due to the reentrant activity being organized around two I-type filaments with endpoints at the epicardium and the infarct scar (Fig. 3B, pink lines). Since the filaments did not extend to the endocardium, no rotational activity was observed there. Both filaments were stably located within the GZ throughout the duration of the simulation.

For all induced VTs in these models, the reentrant waves propagated through the small viable tissue channels in the scar, sometimes with the appearance of an apparent reentry (Fig. 3C). However, in such instances, the behavior was simply wave propagation around an obstacle (the latter part of the scar) and was not sustained. Thus, the reentrant activity underlying the monomorphic VT was driven by reentry filaments that were always located, in their entirety, within the bulk GZ.

### Role of GZ size in VT Morphology

**Figure 4:**
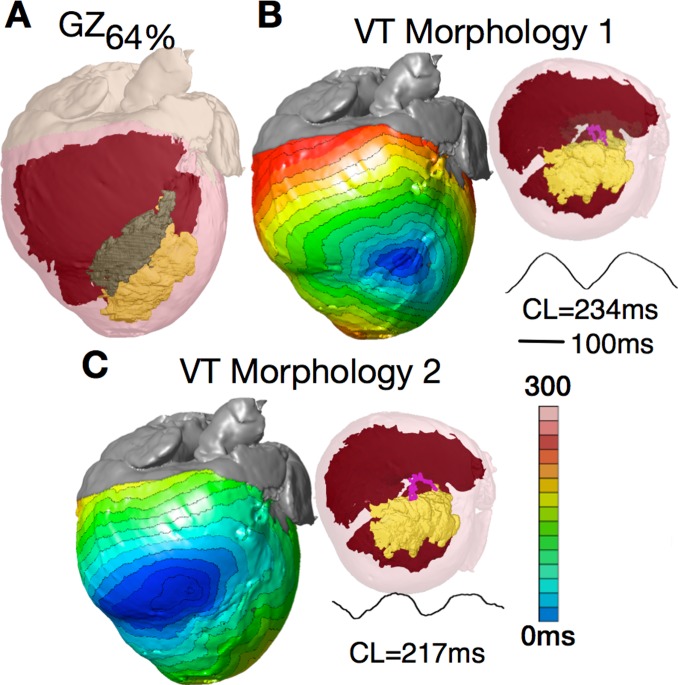
Morphological erosion of GZ to obtain a smaller volume. (**A**) Model with GZ volume 64% of the original. The GZ no longer extends to the epicardium. (**B** and **C**) Epicardial activation maps of the two VT morphologies induced after PES; both manifest as breakthrough on the epicardium and are organized around a U-type filament with endpoints on the scar. The pseudo-ECGs associated with both VT morphologies are also shown.

As stated in the Methods, since dog hearts exhibit a more extensive epicardial GZ compared to humans, we created additional ventricular models by decreasing GZ volume to represent values reported in arrhythmogenic human hearts. All decreases in GZ volume implemented here (see Methods) resulted in the GZ becoming intramural and no longer extending to the epicardium as in the original canine ventricular model. In the model with GZ at 64% of the original volume (Fig. 4A, GZ = 3.23 cm^3^), PES from the same 27 endocardial sites induced 9 VTs (average cycle length 227±23 ms) with two distinct pseudo-ECG morphologies (Fig. 4B–C). For both VT morphologies, the VT manifested itself as a breakthrough on both endo- and epicardium (Fig. 4B–C), with a figure-of-eight intramural pattern. In both cases, the reentrant activity was organized around a single U-type filament attached with both ends to the scar and fully contained within the GZ. However, the position of the U-filament and the locations of the breakthrough sites were different for the two distinct VTs.

Further reduction of GZ to 37% of the original volume (1.88 cm^3^) resulted in VT induction by PES from 7 sites with an average VT cycle length of 196±7 ms; all VTs had the same morphology. VT was similarly organized around a U-type filament located in its entirety within the GZ, which remained stable for the duration of the simulation. Consistent with spiral wave behavior organized around a U-type filament attached to an intramural boundary, reentry was again intramural with breakthroughs on both epi- and endocardial surfaces.

Further morphological erosion of GZ resulting in critical GZ volume of 15% of the original (0.76 cm^3^) resulted in inability to induce VT from any pacing site. In this case, the GZ volume was not large enough to support the formation of stable filaments. No sustained VT could be induced at any values of GZ volume below this critical value. These results indicate that there is a minimum GZ volume necessary in order to support the formation of reentry filaments. This result also explains why filaments cannot be formed in the viable tissue channels within the scar.

### Sensitivity of VT Filaments to GZ Heterogeneity

The simulation results described above clearly demonstrate the paramount role that the GZ plays in establishing the locations, number, and type of the scroll-wave filament(s) that sustain monomorphic VT in the post-MI heart. The next set of simulations examined the contribution of the heterogeneous nature of the GZ to VT morphology.

**Figure 5:**
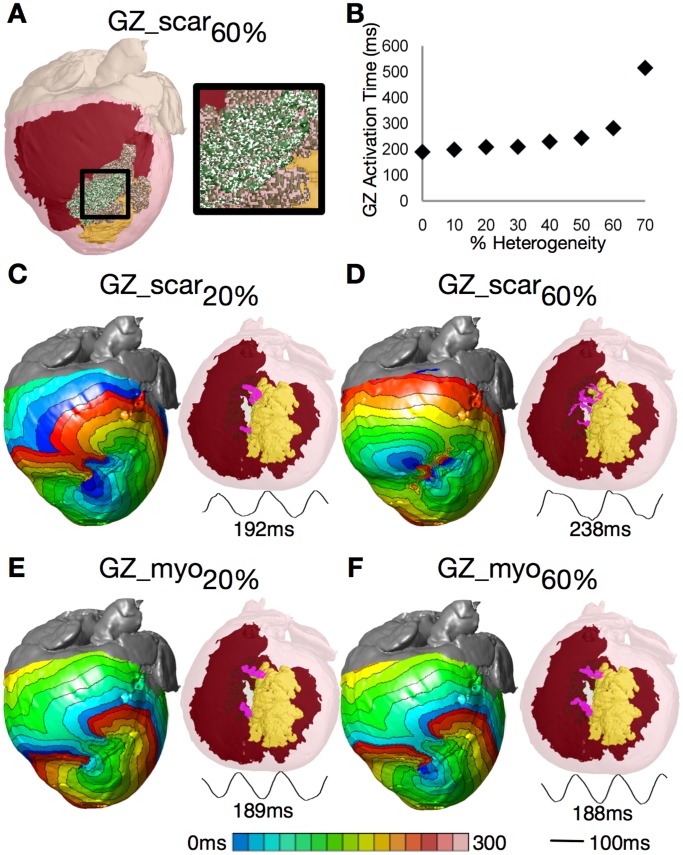
Sensitivity of filament position to GZ electrophysiological properties. (**A**) Model with 60% scar in GZ (white speckles). (**B**) Time needed to fully activate GZ by propagation as a function of scar density in GZ. (**C**) and (**D**) VT activation maps, filaments, and pseudo-ECGs for GZs composed of 20% and 60% scar. (**E** and **F**) Same for GZ composed of 20% and 60% normal myocardium.

In the first set of simulations, micro-regions of scar were randomly distributed throughout the GZ volume at varying densities (see Methods). Following PES, the locations of the resulting filaments were compared to those in the corresponding homogeneous GZ model. Incorporation of micro-regions of scar in the GZ (Fig. 5A shows the 60% case) resulted in conduction slowing within the GZ. The total time it took to fully activate the GZ increased with increased degree of scar density (Fig. 5B). For the heterogeneous cases where GZ was composed of up to 40% scar, the maximum value of scar infiltration as found in histological studies [18], all induced VT morphologies were identical to the control (Fig. 3B). Figure 5C shows the activation maps and filament locations for the model that incorporated 20% scar in the GZ. VT cycle length was 2% longer than in control, with VT again manifested as a figure-of-eight reentry on the epicardium and breakthrough on the endocardium. Most importantly, the I-type filaments remained in the same spatial position.

As GZ scar density increased to more than 70%, wavefronts did not fully propagate through GZ, rendering it functionally identical to the necrotic scar; VT was also not inducible. In Fig. 5D (60% scar), VT cycle length was 26% longer than in control. The VT was manifested as 6 reentries on the epicardium, with multiple filaments densely packed within the GZ. Despite the more complex VT spatiotemporal dynamics, filaments remained within the same general area as in control.

In the second set of simulations, we similarly incorporated random micro-regions at increasing density in the electrophysiologically-remodeled GZ, but this time the micro-regions were composed of normal myocardium. The simulations revealed that models with unchanged GZ conductivities but GZ composition incorporating up to 80% normal tissue exhibited the same VT morphology as in control; VT cycle lengths also did not differ significantly from the control (188.1±0.76 ms). Increasing the amount of normal tissue in GZ to 90% and 100% rendered VTs non-inducible. Fig. 5E–F shows the activation maps and filament locations for the VTs induced in models with 20% and 60% normal tissue in GZ. In both cases, there were slight changes in the activation pattern within the GZ as compared to control, but the reentrant patterns remained of the same type.

## Discussion

In this work, we successfully developed a highly detailed computational model of the infarcted canine ventricles. Previous efforts in modelling of infarcted hearts have been limited to 2D representations [46] or lacked full characterization of the entire ventricles including specific fiber orientations [47]. The individualized infarcted canine ventricular model used in this study incorporated accurate geometry, infarct distribution, and fiber orientation obtained from high resolution ex-vivo MRI and DTMRI. Simulations with this computational model, where VTs were induced following a clinical PES protocol from numerous pacing sites provided novel mechanistic insight into the relationship between infarct-related VTs and the GZ in the post-MI heart. The baseline canine ventricular model was also used to generate additional ventricular models with different GZ sizes, as well as models in which the GZ was represented as different heterogeneous combinations of viable tissue and necrotic scar. To determine whether the GZ is the perpetrator in maintaining infarct-related VT, the organizing centers of VT, the scroll-wave filaments, were calculated and their locations with respect to the infarction zone components (scar and GZ) were determined. We also determined whether changing the size of the GZ while maintaining its shape altered VT morphology as well as the location, number and type of the scroll-wave filaments, and whether similar changes took place when GZ heterogeneity was progressively increased.

### Organizing Centers of Infarct-related VT Reside in the GZ

The results of the VT induction simulations with a number of high-resolution canine ventricular models (22 altogether) demonstrated that the GZ was the critical factor resulting in VT induction and maintenance. In all inducible models, the VT scroll-wave filaments were contained entirely within the GZ, regardless of GZ size or level of heterogeneity of its composition. GZ was thus the arrhythmogenic substrate that promoted wavebreak and reentry formation. While the necrotic scar played a role in determining the reentrant pathway, GZ always contained the VT organizing centers; all induced VTs were thus of both structural and functional nature.

Our simulations also showed that there is a minimum volume of GZ required to render post-MI hearts arrhythmogenic. The critical GZ size obtained in our simulations is comparable with those determined in experiments. Estner et al reported that pig hearts with inducible VTs had a mean GZ volume of 1.62±.66 cm^3^, while hearts that were non-inducible had a mean GZ volume of 0.62±0.20 cm^3^ [14]. These values match with those obtained in our simulations where hearts with GZ volume more than 0.76 cm^3^ were found to be inducible. Our results further demonstrate that large GZ volumes were able to support a larger number of stable filaments, resulting in multiple VT morphologies arising from the same infarct geometry (Figs. 3–4). Intermediate GZ volumes were able to support typically a single filament, giving rise to the same VT morphology regardless of the PES site, while GZ volumes below the critical value resulted in VT non-inducibility due to insufficient amount of electrically remodelled tissue to support reentrant activity. The shape of the filaments transitioned from I-type to U-type (attached to the endocardium) to U-type (attached to the scar) as GZ size progressively decreased, with GZ becoming fully intramural.

While figure-of-eight reentry have been widely described within the extensive epicardial GZ (border zone) in canine post-MI hearts (with which our simulation results are consistent, Fig. 3) and shown to be sustained by the electrophysiological remodeling in GZ [28], [48]–[50], the relationship between infarct zones and VT circuits in species with much smaller, mostly intramural GZs (such as pig or human) has remained controversial, with structural remodeling and fibrosis postulated as the possible reentry substrate [51]–[53]. New compelling evidence has recently shown that structural remodeling alone is inadequate in explaining the mechanisms of post-MI VT. The study by Greener et al showed that post-MI pigs that are inducible for VT are characterized with decreased Cx43 expression compared to pigs that are non-inducible [48]. Since the GZ is the region that is characterized with decreased expression and increased lateralization of Cx43, as demonstrated by molecular studies [28], [54], [55], the Greener et al [48] findings implicate the GZ in VT inducibility. Additionally, it was shown that gene transfer of Cx43 into the GZ of arrhythmogenic pigs resulted in increased levels of Cx43, increased conduction velocity, and decreased arrhythmia inducibility.

Our finding that even for the small GZ sizes typical of human infarction, the GZ is the region maintaining the VT organizing centers is also corroborated by recent clinical evidence. Yan et al analyzed the extent of GZ by LGE-MRI as a predictor of mortality in patients with evidence of MI [10]. After a follow-up of 2.4 years, patients with an above-median percentage of GZ were statistically at a higher total mortality risk (28%) compared with those with a lower percentage of GZ (13%). Similarly, Schmidt et al studied patients with history of MI referred for ICD implantation for primary prevention of sudden death [12]. Patients with induced sustained monomorphic VT had similar infarct sizes but a larger GZ extent than the noninducible patients (19±8 vs 13±9 g, respectively). In their logistic regression analysis, the GZ was the only variable significantly related to VT inducibility (neither infarct location nor core extent was).

Our finding that GZ contains all the VT scroll-wave filaments has an important clinical significance. It supports promising new use of MRI to evaluate the arrhythmia risk of patients with coronary artery disease, assessing the GZ extent from the clinical scans. The potential advantages of such an approach are that it is a noninvasive technique, it is applicable to a wide range of patients, and the reproducibility of the image data is high. Furthermore, one would envision an even more targeted patient-specific approach to the assessment of infarct-related arrhythmia risk, in which computer simulations with in-vivo clinical-MRI-based computer model of the infarcted patient heart (with reconstructions of both scar and GZ, similar to the model and simulations presented in this study) would be used to determine noninvasively whether VT is inducible in the patient heart, the latter then warranting an ICD implantation. Initial attempts in this direction have already been made [47], although there were significant limitations in the simulation approach.

### Organizing Centers of Infarct-related VT are Insensitive to Structural Heterogeneities in GZ

Understanding the relationship between the VT organizing centers and GZ has also important clinical implications for infarct-related VT ablation since it could provide guidance in determining the optimal targets of VT ablation. We envision that patient specific computer simulations, such as the ones presented here, could be used to determine the locations of the VT organizing centers; these locations would then be targeted for ablation. This will pave the way for a major shift in the clinical procedure of VT ablation, where accurate identification of the optimal targets of ablation in each patient heart will be carried out non-invasively by simulation methodology prior to the clinical procedure. This could result in a significant improvement in the efficacy of and tolerance for the therapy.

We found that the VT scroll-wave filament locations are not particularly sensitive to the structural composition of the GZ and are determined predominately by GZ morphology and size. Our simulations show that the presence of up to 40% of scar in the GZ (the maximum scar infiltration of GZ as per histological evidence [13]) does not affect the filament spatial position; even with 70% scar in GZ, the induced VTs still had filaments located in approximately the same general region as in the model without scar tissue in GZ (Fig. 5C–D). For a given PES site, GZ morphology and size were found to be the main determinants of filament location, number, and type. Our simulations demonstrated that approximating the GZ as a homogeneously remodelled tissue with slowed conduction is sufficient to predict the locations of post-MI VT filaments. This finding is very important for the potential translational efforts in developing simulation predictions for the optimal targets of VT ablation. It makes the clinical translation of the approach feasible because it eliminates the need to obtain high-resolution information about the GZ structural properties in each individual heart, allowing for patient-specific models of infarct-related VT could employ a simplified homogeneous representation of the GZ.

### Limitations

The present study employed an MRI/DTMRI-based model of the canine ventricles. While the size of the GZ was decreased from the original extensive epicardial GZ to sizes typical for the human in an additional set of models, the geometry of the heart and the infarct scar is that of a canine heart. Additional studies need to be performed to confirm the findings of this study in the human heart.
